# Mutant K-*ras* oncogene regulates steroidogenesis of normal human adrenocortical cells by the RAF-MEK-MAPK pathway

**DOI:** 10.1038/sj.bjc.6600589

**Published:** 2002-10-21

**Authors:** C-H Wu, Y-F Chen, J-Y Wang, M-C Hsieh, C-S Yeh, S-T Lian, S-J Shin, S-R Lin

**Affiliations:** Graduate Institute of Medicine, Kaohsiung Medical University Kaohsiung, 80317, Taiwan; Department of Laboratory Medicine, Kaohsiung Medical University Kaohsiung, 80317, Taiwan; Graduate Institute of Biochemistry, Kaohsiung Medical University Kaohsiung, 80317, Taiwan; Internal Medicine, Kaohsiung Medical University Kaohsiung, 80317, Taiwan

**Keywords:** K-*ras* mutants, functional adrenal tumour, normal adrenocortical cells, cortisol secretion, RAF, MEK

## Abstract

The result of our previous study has shown that the K*-ras* mutant (*pK568MRSV*) transfected human adrenocortical cells can significantly increase cortisol production and independently cause cell transformation. The aim of this study is to investigate the effect of the active K*-ras* oncogene on the cortisol production in normal human adrenocortical cells. First we used isopropyl thiogalactoside to induce the inducible mutant K-*ras* expression plasmid, *pK568MRSV,* in the stable transfected human adrenocortical cells. The result showed that the increase of RasGTP levels in transfected cells was time-dependent after isopropyl thiogalactoside induction. Additionally, results from Western blot analysis revealed significant elevation in phosphorylation of c-Raf-1 and Mitogen-activated protein kinase. We also detected the levels of mRNA encoding Cholesterol side-chain cleavage enzyme (P450_SCC_), 17α-Hydroxylase/17,20-lyase (P450_c17_) and 3β-Hydroxysteroid dehydrogenase (3βHSD) were increased in human adrenocortical cells transfected with mutant K-*ras* after IPTG treatment. The increase of mRNA amount in P450_scc_ P450_c17_ and 3βHSD and the elevation of cortisol level were inhibited with a pretreatment of PD098059, a specific extracellular signal-regulated kinase inhibitor. In our previous report, we proved that lovastatin, a pharmacological inhibitor of p21^ras^ function, also reversed the increase of cortisol level in mutant K-*ras* stably transfected human adrenocortical cells. Taken together, these findings proved that the active mutant Ras enhanced not only cell proliferation but also steroidogenesis in steroidogenic phenotype cells by activating Raf-MEK-MAPK related signal transduction pathway. Therefore, we believe that K-*ras* mutants influence regulation of steroidogenesis in adrenocortical cells through RAF-MEK-MAPK pathway.

*British Journal of Cancer* (2002) **87**, 1000–1005. doi:10.1038/sj.bjc.6600589 www.bjcancer.com

© 2002 Cancer Research UK

## 

Our laboratory has been studying human functional adrenocortical tumours since 1993 (Lin *et al*, 1994, 1996, 1998a,b, 2000; Shu *et al*, 2001; Wu *et al*, 2002). We have found that the K-*ras* gene enhanced the efficiency of cell proliferation and elevation of cortisol production in bovine adrenocortical cells. However, it did not independently induce cell transformation (Shu *et al*, 2001). To increase the credibility of our experiments, we cultured normal human adrenocortical cells from donors for mutant K-*ras* gene analysis. The cultured normal human adrenocortical cells synthesised and secreted steroid hormones. The levels of cortisol and aldosterone indicated that the cultured cells were mainly fasciculata reticularis cells with very few contaminating glomerulosa cells (Wu *et al*, 2002). The results also showed that cortisol levels of the K*-ras* mutant (*pK568MRSV*) transfected human adrenocortical cells were 18 to 25 times higher than that in controls. In addition, the mutant K-*ras* gene could induce morphologic alterations in the transfected adrenocortical cells (Wu *et al*, 2002). These studies proved that the mutant K-*ras* gene takes part in steroidogenesis of human functional adrenocortical cells. Nevertheless, it is not quiet clear whether the mutant K-*ras* gene enhances steroidogenesis through Mitogen-activated protein kinase (MAP kinase, MAPK) pathway or others.

The core components of the MAPK signalling cascades are three sequential kinases, including MAP kinase (MAPK, or extracellular signal-regulated kinase, ERK), MAPK kinase (MAPKK, or MAPK/ERK kinase, MEK), and MAPKK kinase (MAPKKK, or MEK kinase, MEKK). There are three characterised MAPK signalling pathways: (1) the Ras-to-MAPK signal transduction pathway (or ERK pathway), which is responsive to signals from receptor tyrosine kinase and some heterotrimeric G-protein-coupled receptors, which promote cell proliferation or differentiation. Ras acts as a molecular switch by cycling between active GTP-bound and inactive GDP-bound states. The active RasGTP form transfers the signal to Raf. The active Raf will phosphorylate MEK and then the phosphorylated MEK will phosphorylate ERK1/2(MAPK). Consequently, the phosphorylated ERK1/2 will activate a series of target proteins such as the ribosomal protein S6 kinases (RSKs), E1k-1, SOS, STATS, and C-Myc, which lead to consequent reactions (Treisman, 1996; Fanger *et al*, 1997; Elion, 1998); (2) the stress-activated protein kinase/c-Jun N-terminal kinase (SAPK/JNK) pathway; and (3) the p38 pathway (Robinson and Cobb, 1997; Garrington and Johnson, 1999). Many inhibitors are used to stop signal transduction in Ras/MAPK pathway research. One of them, PD098059, a synthetic inhibitor of MEK (MAPKK) has high specificity and has been used by other researchers. Some studies have indicated that PD098059 failed to inhibit the stress, the stimulated JNK/SAPK and the p38 pathways, demonstrating its specificity for the ERK pathway (Alessi *et al*, 1995; Dudley *et al*, 1995).

Interestingly, most patients with functional adrenocortical tumours sought hospital treatment not owing to the pain caused by the cancer but owing to the physiological imbalance caused by excess secretion of cortisol. Therefore, the influence of hormone secretion seems to be more significant than that of cell growth regulation in adrenocortical tumours. In the past, research regarding cholesteroid regulation has often focused on the well known regulation of elevated caused by adrenocorticotropic hormone (ACTH) and Angiotensin II (Fredlund *et al*, 1975; Roskelley and Auersperg, 1995; Toaff *et al*, 1979). Little is known concerning the association hormone secretion of oncogenes with secretion regulation. Based on our previous results, we assumed that the active K-ras oncogene induced sterogenesis in adrenocortical cells through Ras-to-MAPK pathway. To prove our assumption, we used the mutant K-ras to activate the RAF/MEK (extracellular signal-related kinase kinase)/MAPK/ERK pathway. In order to understand whether mutant K-Ras activated C-Raf and MAP kinase, we transfected the *pK568MRSV*, the inducible mutant K-ras oncogene expression plasmid, into normal human adrenocortical cells (Wu *et al*, 2002). Then we analysed the level of RasGTP during the extension time after IPTG treatment and the level of phosphorylated c-Raf-1 and MAP kinase by Western blot. We used a specific MAPK kinase (MEK) inhibitor, PD098050, to inhibit signal transduction to observe whether mutant K-ras induced on increase of mRNA levels of Cholesterol side-chain cleavage enzyme (P450_SCC_), 17α-Hydroxylase/17, 20-lyase (P450_c17_) and 3β-Hydroxysteroid dehydrogenase (3βHSD) and the cortisol levels in transfected cells. By doing this, we hope the mechanism of the K-ras oncogene taking part in steroidogenesis will be further understood.

## MATERIALS AND METHODS

### Mutant K-*ras* gene stably transfected human adrenocortical cell culture

The cultured normal human adrenocortical cells synthesised and secreted steroid hormones. The levels of cortisol and aldosterone secretion indicated that the cultured cells were mainly fasciculata reticularis cells with very few contaminating glomerulosa cells as described previously (Wu *et al*, 2002). Cells (2×10^5^) in a 35-mm plate were transfected with *pOPRSV* (control vector) and *pK568MRSV* *K-ras* expression plasmids by CLONfectin method (BD Biosciences Clontech, Palo Alto, CA, USA). Cells to be transfected were removed from the cultures and the CLONfectin/DNA media solution was gently applied. The plates were incubated at 37°C for 1–4 h in a CO_2_ incubator. CLONfectin/DNA- media solution was removed and then cells were washed with medium or 1× phosphate buffer saline (PBS). Fresh complete growth medium was added and the plates were incubating for another 48 h. Then the growth medium was changed to the medium containing 400 μg ml^−1^ G418 (BD Biosciences Clontech) and changed every 2 to 3 days to select the cell monoclone containing expression plasmid.

### Construction of GST-RBD of Raf-1 expression plasmid

Using the PCR we constructed the expression plasmid for minimal Ras-binding domain of human Raf-1 (aa 51–131) from the human liver Marathon-ready cDNA (BD Biosciences Clontech, CA, USA). The oligonucleotides used to amplify a fragment of human c-raf-1 were 5′-CACAGATGGATCCAAGACAAGCAACAC-3′ and 5′-GGGAAGAAT-TCACAGGAAATCTAC-3′ (Herrmann *et al*, 1995) cDNA. The resulting 412-base pair fragment was cleaved with *Bam*HI and *Eco*RI, purified, and cloned into the GST fusion vector pGEX-4T-2 (Amersham, Pharmacia Biotech UK, Ltd) as described previously (Lin *et al*, 2000). The resulting plasmid, pGEX-RBD, was transformed into *E. coli* strain TG1. The correct orientation of the RBD of Raf-1 cDNA was confirmed by sequencing.

### Expression and purification of GST-RBD of Raf-1 fusion protein

The expression of fusion protein was performed basically as described by Guan and Dixon (1991). Twenty millilitres of an overnight culture was inoculated into 1 l of 2XYT (10 g yeast extract, 16 g tryptone and 5 g sodium chloride (NaCl) in 1 l) containing 50 μg ml^−1^ ampicillin. The culture was incubated vigorously at 37°C until an absorption of one at 600 nm was reached. IPTG was then added and cells were harvested by centrifugation and suspended in 10 ml PBST (2 mM EDTA, 0.1% β-mercaptoethanol, 0.2 mM PMSF and 5 mM benzamidine) after 3 h incubation. Bacterial lysate was centrifuged at 4°C to remove the insoluble fraction. One millilitre of the bacterial supernatant containing soluble proteins was mixed with 2 ml 50% (v v^−1^) glutathione agarose beads and incubated 30 min at 4°C with gentle shaking. The agarose beads were washed four times with 10 ml PBST. The fusion protein was eluted by competition with glutathione using 2×2 min washes with 1 ml of 50 mM Tris (pH 8.0) containing 10 mM glutathione. Free glutathione was removed by dialysis against 50 mM Tris (pH 8.0), 150 mM NaCl, 0.1% β-mercaptoethanol. The purified GST-RBD of Raf-1 protein was recovered.

### Precipitation and immunoblot of active form RasGTP by GST-RBD of Raf-1 fusion protein

The desired amount of crude GST-RBD of Raf-1 fusion protein was incubated with glutathion-agarose beads at room temperature for 30 min. The beads were isolated by centrifugation and washed three times with RIPA buffer. Mutant K-*ras* gene stably transfected human adrenocortical cells were grown in Ham's F-12 medium containing 10% foetal calf serum with 2.5 mM IPTG, and cells from a 9 cm dish were lysed and scraped in 1 ml of RIPA buffer containing 50 mM Tris pH 8.0, 150 mM NaCl, 0.5% doc, 1% NP40, 0.1% SDS, 0.1 μM aprotinin, 1 μM leupeptin and 1 mM PMSF at 4°C. Lysates were centrifuged at 14 000 r.p.m for 8 min in an eppendorf centrifuge to remove nuclei. GST-RBD, precoupled to glutathion-agarose-beads in RIPA buffer, was added and the lysates were incubated at 4°C for 30 min. Beads were collected by centrifugation, washed three times with RIPA buffer and resuspended in sample buffer (10% glycerol, 60 mM Tris, pH 6.8. 2% SDS. 300 mM β-mercaptoethanol). The protein samples were separated on vertical slab gels composed of 10% polyacrylamide containing 0.1% sodium dodecyl sulphate (SDS) prepared in 3 mM Tris-HCl, pH 8.8, as described previously (Lin *et al*, 2000). Following electrophoresis, the protein was electroblotted to a nitrocellulose membrane (Schleicher & Schusll GmbH, Dassel, Germany). The membrane fixed with protein was incubated with mouse monoclonal antibodies (mAbs) specific for the K-r*as* p21 product (25 mg ml^−1^) (Santa Cruz Biotechnology, Inc.), and the excess unbounded antibodies were then washed off. The membrane binding with the mouse mAbs was incubated with a solution of secondary goat anti-mouse IgG conjugated with alkaline phosphatase (1 : 3000, Bio-Rad, Hercules, CA, USA) The Ras protein was detected by enhanced chemiluminescence (ECL) detection system (Amersham Pharmacia Biotech UK, Ltd). Blot was then exposed to Kodak XAR film (Eastman Kodak, Rochester, NY, USA).

### Western blot

The protein extracts, obtained from mutant K-*ras* gene stably transfected human adrenocortical cell, were purified and electrophoresed through vertical slab gels as described above. Briefly, following electrophoresis, the proteins were transferred to a nitro-cellulose membrane by an electrophoretic gel transfer device. The membrane fixed with proteins was incubated with mouse monoclonal antibodies specific for the c-Raf-1, phosphorylated raf-1 (p-raf-1), MAPK, phosphorylated MAPK (p-ERK, E-4) (Santa Cruz Biotechnology Inc., Santa Cruz, CA, USA), respectively. The membrane binding with the mouse mAbs was incubated with a solution of secondary goat anti-mouse IgG conjugated with alkaline phosphotase (1 : 3000, Bio-Rad, Hercules, CA, USA) The target proteins were detected by enhanced chemiluminescence (ECL) detection system (Amersham. Pharmacia Biotech UK Ltd). Blot was then exposed to Kodak XAR film (Eastman Kodak, Rochester, NY, USA).

### PD098059 MAPK Kinase inhibitor treatment

The *pK568MRSV* transfected adrenocortical cells were treated with 10 mM PD098059 for 45 min and then 2.5 mM IPTG was added. The mixture was incubated for 36 h. The PD098059/IPTG-treated cells were then ready for cortisol level detection and MAPK kinase assay. The PD098059/IPTG-treated cell lysates and control cell lysates were immunoprecipitated with anti-ERK monoclonal antibody (Santa Cruz Biotechnology Inc., Santa Cruz, CA, USA). The immunocomplexes were then ready for MAP kinase assay.

### MAP kinase assay

Proteins were obtained from *K-ras* expression plasmids transfected cells and control cells. Supernatant (0.5 ml) containing 0.5 mg protein was incubated with 10 μl of goat anti-ERK antibodies (Santa Cruz Biotechnology Inc., Santa Cruz, CA, USA) for 2 h at 4°C. The immunocomplexes were precipitated by centrifugation and washed two times with buffers A, B (500 mM LiCl, 100 mM Tris, 1 mM DTT, 0.1% Triton X-100; pH 7.6), and C (20 mM MOPS, 2 mM EGTA, 10 mM MgC1_2,_ 1 mM DTT, 0.1% Triton X-100; pH 7.2), respectively ERK activities in the immunocomplexes were measured (Alessi *et al*, 1995). Immunocomplexes were incubated with 35 μl of buffer C supplemented with myelin basic protein (MBP; 6 μg; Upstate Biotechnology), [γ-^32^P]ATP (5 μCi), and MgCl_2_ (50 mM) for 20 min at 37°C with vortexing every 5 min. To stop the reaction, 15 μl of 4× Laemmli buffer was added, and the mixture was boiled for 5 min. Proteins in the kinase reaction were resolved by SDS–PAGE (15% gel) and subjected to autoradiography.

### Northern blot

Total RNA was extracted with acid-guanidium phenol/chloroform assay (AGPC) from transfected cells. Twenty micrograms of total RNA was denatured with 6.5% formaldehyde and 50% formaldehyde for 15 min at 55°C and separated by electrophoresis on 1.2% agarose gels containing 1.1% formaldehyde. Then RNA was transferred to nylon membrane (Schleicher and Schuell GmbH, Dassel, Germany) and fixed. The blots were successively hybridised with human P450_SCC_ cDNA, human P450_C17_ cDNA, 3βHSD cDNA and human β-actin cDNA. The hybridised filters were then washed twice with washing buffer (Solution I: 3×SSC, 0.5% SDS; Solution II: 0.5×SSC, 0.1% SDS) at 65°C to wash off the unbounded probe. The membrane was exposed to Kodak X-film at –70°C for autoradiography. β-actin signal was used to normalise data for the above mRNA. All of the probes were confirmed by direct sequencing.

### Cortisol level detection

The cell clones containing stable expression vector DNA were cultured in a 6 mm culture plate. The supernatant was removed for cortisol level detection. Standard solutions were diluted into different concentrations (0, 1, 3, 10 and 60 μg dl^−1^), then 10 μl of each standard solution and cell supernatant were each added into a 96-well plate in which each well contained glass beads coated with anti-cortisol IgG. An enzyme immunoassay assay and a UBIMAGIWEL cortisol QUANTITATIVE kit (United Biotech Inc., CA, USA) were used to detect and quantify the cortisol levels. One hundred millitres of cortisol enzyme conjugate solution was added into each well, mixed with the mixture, and then placed at room temperature (22–27°C) for 60 min. Next, the well was washed with distilled water five times, followed by the addition of solutions A and B, and mixed thoroughly and incubated at room temperature for 30 min. A 50 μl stop solution (1 N H_2_SO_4_) was then added to stop the reaction and the results were qualified using an ELISA processor II (Boehring, Germany) at 450 nm. Finally, the cortisol levels were measured according to the standard curve in μg per 10^6^cells.

## RESULTS

**Figure 1:**
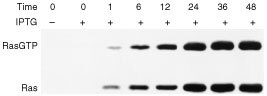
Time course of RasGTP formation after treatment with IPTG. The normal human adrenocortical cells transfected with *pK568MRSV* plasmid were induced with 2.5 mM IPTG. Then the active RasGTP form was precipitated by GST-RBD of Raf-1 fusion protein and immunoblotted as described in Materials and Methods. The RasGTP levels were detected at 0, 1, 6, 12, 24, 36 and 48 h after IPTG induction. Upper panels show the amount of RasGTP; lower panels show total amount of Ras in 10% of the extract. The amount of RasGTP was increased with the elongation of IPTG induction. Plus (+) indicates when present; minus (−) indicates when absent.

**Figure 2:**
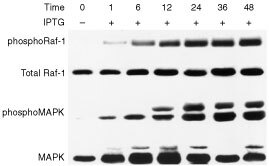
Time course of the phosphorylation of c-Raf1 and MAPK in *pK568MRSV* transfected adrenocortical cells after treatment with IPTG. Crude protein extract from transfected cells was electrophoresised and transfered to PVDF as described in Materials and Methods. And then, it was immunobloted with monoclonal antibodies specific to phosphorylated c-Raf1 (p-Raf-1), phosphorylated MAPK and control c-Raf-1 and MAPK, respectively. The amounts of phosphorylated c-Raf-1 (p-Raf-1) and phosphorylated MAPK; and control MAPK and c-Raf-1 were detected at 0, 1, 6, 12, 24, 36 and 48 h after IPTG induction. The amounts of phosphorylated c-Raf-1 and MAPK were increased with the elongation of IPTG treatment. Plus (+) indicates when present; minus (−) indicates when absent.

The normal human adrenocortical cells transfected with *pK568MRSV* plasmid were induced with 2.5 mM IPTG., and the active RasGTP form was precipitated by GST-RBD of Raf-1 fusion protein and immunoblotted as described in Materials and Methods. The RasGTP levels were detected at 0, 1, 6, 12, 24, 36 and 48 h after IPTG induction (Figure 1

). The results showed that RasGTP levels in cells transfected with *pK586MRSV* increased with induction time. The phosphorated c-Raf-1and phosphorated MAPK levels increased significantly at 1, 6, 12, 24 and 36 h after IPTG induction. The results of this Western blot analysis also revealed that phosphorylation of c-Raf-1 and MAPK was significantly enhanced when RasGTP level increased (Figure 2

). Thus the inducible mutant K-*ras* expression plasmid transfected adrenocortical cells expressed the mutant RasGTP in a time-dependent manner after IPTG induction. Concurrently, the amount of phosphorylated c-Raf and phosphorylated MAPK increased.

**Figure 3:**
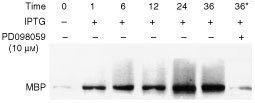
The effect of PD098059 pretreatment and the time course of MEK activity in *pK568MRSV* transfected adrenocortical cells after treatment with IPTG. Kinase activity was measured using myelin basic protein (MBP) as a substrate as described in Materials and Methods and significantly increased at 6, 12, 24 and 36 h after IPTG induction. *The 36* lane represented the *pK568MRSV* transfected cells treated with 10 μM PD098059 for 45 min before the addition of 2.5 mM IPTG and then incubation for 36 h in the presence of PD098059 and IPTG. With pretreatment of PD098059, the MEK activity was obviously inhibited in *pK568MRSV* transfected adrenocortical cells after treatment with IPTG. Plus (+) indicates when present; minus (−) indicates when absent.

**Figure 4:**
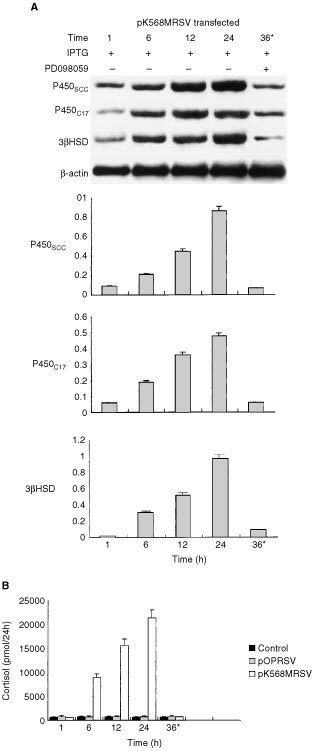
Effect of PD098059 on cortisol production or P450scc, P450c_17_ and 3HSD mRNA in mutant K-*ras* gene transfected human adrenocortical cells. *pK568MRSV* or *pOPRSVI* transfected adrenocortical cells were treated with IPTG for 1, 6, 12, 24 and 36 h. Then the total RNA was extracted from the treated cells for Northern blot analysis. The histogram revealed the densitometry data of Northern blot of p450scc, p450c17 and 3HSD at every time point. The data indicates that their mRNA levels increased with incubation time, however, the increase was blocked by PD69859 as shown in lane 36*. Cortisol level in culture medium was detected by EIA and was found to increase with the elongation of IPTG induction. And the elevated level of cortisol was blocked shown in (**B**) the 36* column. (**B**) *The 36* lane represented the *pK568MRSV* transfected cells treated with 10 μM PD098059 for 45 min before the addition of 2.5 mM IPTG and then incubation for 36 h in the presence of PD098059 and IPTG. Plus (+) indicates when present; minus (−) indicates when absent.

Furthermore, the kinase activity of MEK (MAPK kinase) was measured using myelin basic protein (MBP) as a substrate and significantly increased at 6, 12, 24 and 36 h after IPTG induction (Figure 3

). With pretreatment of 10 mM PD098059 for 45 min, *pK568MRSV* transfected adrenocortical cells were then treated with 2.5 mM IPTG. The mixture solution was incubated for 36 h in the presence of PD098059 and 2.5 mM IPTG. Then the MEK activity was obviously inhibited (Figure 3, lane 36*). Moreover, steroidogenesis study by Northern blot showed that the density of mRNA of p450scc, p450c_17_ and 3βHSD at each period was increased with time, but was also inhibited by PD098059 as shown in Figure 4A

. Thus, inhibition of MEK/MAPK pathway also inhibited these enzymes^,^ synthesis. Consequently, the cortisol production was increased significantly at 1, 6, 12 and 24 h, and the increase of cortisol production was also significantly blocked by PD098059 at 36 h after IPTG induction (Figure 4). By using PD098059, the MEK specific inhibitor, we could confirm that mutant K-*ras* gene enhanced cortisol secretion in normal human adrenocortical cells through the Raf-MEK-MAPK pathway.

## DISCUSSION

The present study showed that the adrenocortical cells transfected with *pK568MRSV*, an inducible mutant K-*ras* expression plasmid, expressed the mutant Ras protein in a time-dependent manner after IPTG induction. At the same time, the amount of phosphorylated c-Raf and phosphorylated MAPK were also increased. To clarify whether mutant K-*ras* gene regulated cortisol levels by MEK/MAPK pathway, we used PD098059, a specific MEK inhibitor (Alessi *et al*, 1995; Dudley *et al*, 1995), to block the signal transdution of the active RasGTP after IPTG induction. PD098059 is a synthetic inhibitor of the Ras-MAPK pathway that specifically blocks the activation of MEK. PD098059 has subsequently been used as a tool to study MAPK signalling in various cell types and in carcinogenesis (Reuter *et al*, 2000, blood). The inhibition of MEK activation was demonstrated to prevent activation of MAPKs (ERK-1/2) and subsequent phosphorylation of MAPK substrates both *in vitro* and in intact cells (Alessi *et al*, 1995; Dudley *et al*, 1995).

In this study, the addition of PD098059 led to the reduction of the accumulation of P450scc, P450c_17_ and 3HSD mRNA after IPTG-induction in transfected human adrenocortical cells by inhibited Ras-MAPK pathway. PD098059 also reversed the increased cortisol level in *pK568MRSV* transfected human adrenocortical cells. In our previous study (Wu *et al*, 2002), we have proved that lovastatin, a pharmacological inhibitor of p21^ras^ function (Roskelley and Auersperg, 1995), also reversed the increased cortisol level in mutant K-*ras* stably transfected human adrenocortical cells. Taken together, these proved that the mutant K-*ras* gene enhanced not only cell proliferation but also steroidogenesis in steroidogenic phenotype cells by activating of Raf-MEK-MAPK signal transduction pathway.

Previous research on regulation of steroidogenesis mostly focused on regulation of elevated hormone secretion caused by ACTH and Angiotensin II (Toaff *et al*, 1979; Roskelley and Auersperg, 1995). Little is known concerning the association of oncogene with steroidogenesis regulation. Our study found that the activation of genes encoding enzymes related to steroidogenesis were regulated by activating cyclic Adenosine 3′,5′ monophosphate (cAMP) regulatory protein to interact with specific sequences while cAMP was used as secondary messenger. These cAMP regulatory proteins included cAMP regulatory element-binding protein (CREB), Steroidogenic factor (SF-1) and sp1 (Morohashi *et al*, 1992; Waterman *et al*, 1992; Omura and Morohashi, 1995). Cytochrome P450scc(CYP11A1) removes the side chain of cholesterol after cholesterol has been transferred into cells by steroidogenic acute regulatory protein (StAP). It is, therefore, also an important rate-limiting enzyme of steroidogenesis process in adrenocortical cells (Miller, 1988). Cholesterol was then used as substrate to produce different kinds of hormones via the action of 3βHSD, P450c_21_ (CYP21B), P450_17a_ (CYP17) and P450 _11b_(CYP11B1). P450 regulatory enzymes were mainly regulated by cis-acting elements on promoter region including cAMP-responsive sequences (CRS), Ad4 and GC-rich sequences binding with corresponding proteins (Morohashi *et al*, 1992; Begeot *et al*, 1993; Guo *et al*, 1994; Tsukiyama *et al*, 1994; Omura and Morohashi, 1995). Meanwhile, steroid 17α-hydroxylase and 3β-dehydrogenase can be regulated by cyclic AMP and protein kinase C secondary messenger system (Lund *et al*, 1997). Among all the positive gene regulations in hormone production, CREB, a highly expressive transcription factor in adrenal cortex, regulated steroid production by binding with frequently present CREB binding site in the promoter region of genes which encoding steroidogenic enzymes (Tsukiyama *et al*, 1994; Chung *et al*, 1997).

In many studies, the K-ras gene has been proven to take part in cell growth regulation by activating MAP kinase to activate transcription factor ELK-1 (Marais *et al*, 1993; Miranti *et al*, 1995). In 1996, Xing *et al* found that introduction of activated *ras* oncogene into PC12 cells regulated gene expression by activating RAF and MAP kinase and then RSK2 of pp90RSK family to phosphorylate CREB (Xing *et al*, 1996; Cesare *et al*, 1998; Beaupre *et al*, 1999). The CREB was the earliest found to regulate gene expression when the level of cAMP increases (Bonni *et al*, 1995). It also participates in the regulation of the cell response after the binding of growth factors with receptors (Bohm *et al*, 1995). Furthermore, CREB is a transcription factor for genes corresponding to steroidogenesis related enzymes (Watanabe *et al*, 1994; Kagawa *et al*, 1999). In this study, our laboratory proved that the mutant Ras protein enhanced not only the cell proliferation but also the steroidogenesis in steroidogenic phenotype cells by activation of Raf-MEK-MAPK signal transduction pathway. Therefore, in the future, we will use the cDNA microarray and other tools to examine the difference between the control cell and the K*-ras* transfected cell to understand the cross talks between the Ras-to-MAPK pathway and the regulation of the steroidogenesis process.
